# Nitrogen Balance in Female Japanese National Handball Players During Training Camp

**DOI:** 10.3389/fnut.2020.00059

**Published:** 2020-05-12

**Authors:** Haruka Suzuki, Yuki Ueno, Toshiya Takanouchi, Hiroyuki Kato

**Affiliations:** ^1^Olympic & Paralympic Promotional Office, Corporate Service Division, Ajinomoto Co., Inc., Tokyo, Japan; ^2^Japan Handball Association, Tokyo, Japan

**Keywords:** protein requirement, nitrogen balance, elite athlete, handball, female athlete, leucine

## Abstract

Protein requirements for athletes are affected by various factors, including distribution and quality (i. e., amino acid composition) of protein ingestion throughout the day. However, little is known about the protein requirements of elite female athletes engaging in team sports. This study aimed to determine the nitrogen balance and distribution of protein and amino acid intake in elite female handball athletes during training camp. In observational study design, 11 female Japanese national handball players (age 26.9 ± 4.9 years) participated in a 5-days experiment. Nitrogen balance was calculated from the daily protein intake assessed by dietary records and urinary nitrogen excretion. Amino acid intake amounts were organized based on six eating occasions. The average and population-safe protein intake for zero nitrogen balance were estimated as 1.57 and 1.93 g/kg/day, respectively. The protein intake at breakfast, lunch, and dinner and the leucine intake in the three main meals and the morning snack were higher than is recommended by current guidelines for maximizing muscle protein synthesis. The population-safe protein intake in elite female handball athletes was within the range of the current recommendations for athletes (1.2–2.0 g/kg/day). Our results show that it may be possible to improve the distribution and quality of protein ingestion after exercise and before sleep.

## Introduction

A higher protein intake (1.2–2.0 g/kg/day) than the current recommended daily allowance (RDA; 0.8 g/kg/day) is recommended for athletes (1). The recommended intake ranges widely because protein requirements are affected by the type, intensity, and duration of exercise, sex, and other nutritional states (energy balance and carbohydrate intake) (2). Since numerous factors can modulate protein requirements, it remains to be elucidated how those various factors can impact protein requirements. In particular, knowledge concerning protein requirements in elite athletes is limited, owing to difficulties in conducting experiments (potential disturbance of training and limited number of potential subjects) with elite athletes. Thus, nutritional guidelines for elite athletes are generally based on self-reported dietary records of elite athletes (3–6). However, it is unclear whether their protein intake meets their needs.

In general, elite athletes have high-volume and high-intensity training loads, reaching 15 h/weeks of training (7). Since amino acid oxidation contributes ~5% of total energy expenditure during exercise (8), the exercise-induced oxidative loss of amino acids increases with increased training volume. In addition, since protein requirements for athletes have generally been set for those engaging in endurance (9–11) or resistance exercise (11, 12), the effect of exercise type rather than endurance or resistance exercise on protein requirements remain unclear. Team sports (e.g., basketball, soccer, and handball) are characterized by patterns of intermittent activity with bursts of high-intensity and “stop-and-go” movements. Although these patterns are assumed to incorporate features of both endurance exercise and resistance exercise, knowledge regarding the protein requirements for such specific types of exercise is limited. Recently, Moore's group reported that the recommended protein intake in physically active subjects engaging in variable-intensity intermittent exercise was 1.71 g/kg/day for women (13) and 1.40 g/kg/day for men (14). However, no studies have investigated the protein requirements of elite team sport athletes.

Several recent sports science consensus statements have reported that the timing or composition of protein or amino acid intake could affect athletes' protein metabolism (15, 16). According to the official position of the International Society of Sports Nutrition (15, 16), the ingestion of 0.25–0.40 g of protein /kg body mass/dose from a high-quality source every 3–4 h appears to maximize the muscle protein synthesis (MPS) rate. Thus, it is important to assess the timing and composition of the amino acids consumed in meals and snacks during the day. Therefore, this study aimed to investigate the protein requirements and recommended protein intake for elite female handball athletes during training camp using the nitrogen balance (NBAL) technique. In addition, we analyzed timing and the amino acid compositions of each meal and snack to compare them with the current guidelines.

## Methods

### Ethics Statement

All participants were informed of the study's purpose, experimental procedures, and all potential risks before written consent was obtained. The informed consent was completed by the participants before any study related procedures were performed. This study was conducted in accordance with the Declaration of Helsinki, and the protocol was approved by the institutional review board of Ajinomoto Co., Inc. (No. 2017-016). This trial was registered at www.umin.ac.jp/ctr/index.htm as UMIN000029896.

### Experimental Design

The experiment was conducted using an observational study design November 2017, during the Japanese national team training camp at Ajinomoto National Training Center. Sixteen participants were recruited from female adults who belonged to Japanese women's national handball team, participated in their training camp November 2017, and aged from 18 to 35 years. The participants were excluded if they had any injury and disease to unable to complete designated training activities. In addition, the data were excluded from the data analysis if the participants failed to complete data collection. The experiment protocol is shown in Supplementary Figure 1. The participants performed training activities provided by the coach of the national team, which contained two types of exercise: handball practice and resistance exercise. On days 1, 2, and 4, two training sessions (morning and afternoon) were held. On day 1 and 4, one of training sessions consisted of handball practice, and the other consisted of resistance training. On day 2, both sessions consisted of handball practice. Resistance exercise comprised exercises such as squat, bench press, hip thrust, and leg press. Menstrual cycle information was obtained by questionnaire as the date of the last menstrual period. To investigate the protein requirements and recommended protein intake for elite female handball athletes during training camp, NBAL was used as primary outcome, and the amino acid compositions of each meal and snacks were used as secondary outcome.

### Nitrogen Balance

The NBAL technique was utilized to determine protein requirements in elite handball athletes in a free-living condition to obtain practical knowledge for recommended protein intake in training camp settings. All urine produced on day 2 after the initial morning spot urine until the first urination on day 3 was collected to determine NBAL. The collected urine was acidified with anhydrous citric acid and stored at 4°C until analysis. Creatinine and urea concentrations were measured by SRL Medisearch, Inc. (Shinjuku, Tokyo, Japan). Nitrogen excretion was estimated using the urinary nitrogen of the urea and creatinine, representing >75% of the total daily nitrogen excretion, according to previously published values in a trained endurance running population consuming 1.7 g/kg/day of protein (11). NBAL was calculated as NBAL = E – I, where E represents nitrogen excretion and I represents total nitrogen intake, calculated as the protein intake on day 2 divided by 6.25—a factor used to convert “g protein” into “g nitrogen.”

### Dietary Intake

On days 1, 2, and 4, all the nutrient intake was assessed by a registered dietitian in our research team. All participants had three main meals (breakfast, lunch, and dinner) at the buffet-style restaurant, where they were accustomed to have meals. Participants were instructed to maintain their usual eating habits. In the restaurant, almost all menus, except for main source of carbohydrate (e.g., steamed rice), were served in standardized sizes. The main source of carbohydrate was weighed using a portable scale. Before participants started eating, a registered dietician checked what they picked. In addition, after they finished eating, the dietician recorded leftover. The intake of all snacks (food, supplements, and beverage) was recorded over the 3 days with detailed descriptions (e.g., weight or sizes, number, brand names, etc.), the timing of consumption, and photos. A registered dietician confirmed further details of the snack by checking each morning during camp.

Based on the assessment by the registered dietician, dietary intake (energy, carbohydrate, protein, and fat) was calculated using Excel Eiyo-kun ver. 8 (Kenpaku-sha, Tokyo, Japan) software that contains nutrition-related information of various foods or combinations of selected foods as well as the amino acid contents of some Japanese foods. To analyze the quantity and timing of each amino acid intake over the day, consumption timing was categorized into six eating occasions: breakfast, morning snack, lunch, afternoon snack, dinner, and pre-sleep snack.

### Energy Expenditure and Energy Balance

The total energy expenditure was calculated from summing the resting energy expenditure (REE), energy expenditure during habitual daily physical activities, diet-induced thermogenesis (DIT), and energy expenditure during training sessions. The REE value was calculated using Nelson's equation as follows: REE (kcal/day) = 25.80 × Fat-free mass (kg) + 4.04 × Fat mass (kg) (17). Participants were required to wear an accelerometer (wGT3X-BT, Actigraph, Pensacola, FL, USA) during the trial (except when bathing and exercising) to monitor their habitual physical activity. The DIT was estimated from the total energy intake and protein-fat-carbohydrate (PFC) ratio in the diet according to previous results (18). A heart rate monitor (M430 or V800, Polar Electro, Kempele, Finland) was provided for participants to wear during handball exercise sessions to monitor the duration and intensity of the exercise. %HRmax was calculated as follows: %HRmax = HR/(220 – age) (19). Energy expenditure during handball practice was calculated from the average heart rate (HR), exercise duration (time), sex, body weight, and age using Keytel's equation (20). In particular, energy expenditure during resistance exercise was estimated based on Phillips' study (21). The energy balance on each day was estimated by calculating the energy intake minus the total energy expenditure.

### Statistical Analysis

Based on the former result (1) and with α = 0.05 and β = 0.8, we calculated *n* = 16 would be sufficient to detect a linear relationship between protein intake and nitrogen balance. To ensure the number of the subjects, we targeted to recruit *n* = 20 because we assumed potential drop-outs in the process of the study.

Values are reported as the mean ± standard deviation (SD). Paired *t*-tests were used to determine differences in body weight and body composition from the first to last day. To determine whether energy balance (energy intake – total energy expenditure) was significantly positive or negative, a paired *t*-test was used to compare differences from zero on days 1, 2, and 4. To determine whether the intake (for protein and leucine) was significantly higher than the recommended intake, a paired *t*-test was used to compare differences from 20 and 0.7 g, respectively, in each meal or snack. Linear regression analyses were applied to NBAL data to determine the estimated average protein intake for achieving zero NBAL and the population-safe protein intake as the upper limit of the 95% confidence interval of the estimated average protein intake using the coefficients of variation (22). Data were analyzed using GraphPad Prism 7 software (GraphPad Software Inc., San Diego, CA, USA); values of *P* < 0.05 were considered significant.

## Results

### Participants

One participant was excluded from the study due to an inability to perform the designated training schedule. In addition, the data from four participants were excluded from data analysis due to failure to collect samples. Thus, eleven participants (age 26.9 ± 4.9 years, height 170.6 ± 7.1 cm, weight 66.5 ± 6.4 kg, fat-free mass 49.4 ± 4.1 kg, fat mass 17.2 ± 2.7 kg) (mean ± SD) completed the experiments. Five and six participants were in the luteal and follicular phases, respectively. Participants' body weight (pre: 66.5 ± 6.4 kg, post: 66.6 ± 6.3 kg), fat mass (pre: 17.2 ± 2.7 kg, post: 17.0 ± 2.6 kg) and fat-free mass (pre: 49.4 ± 4.1 kg, post: 49.7 ± 4.0 kg,) did not change over the experimental period.

### Nitrogen Balance

**Figure 1:**
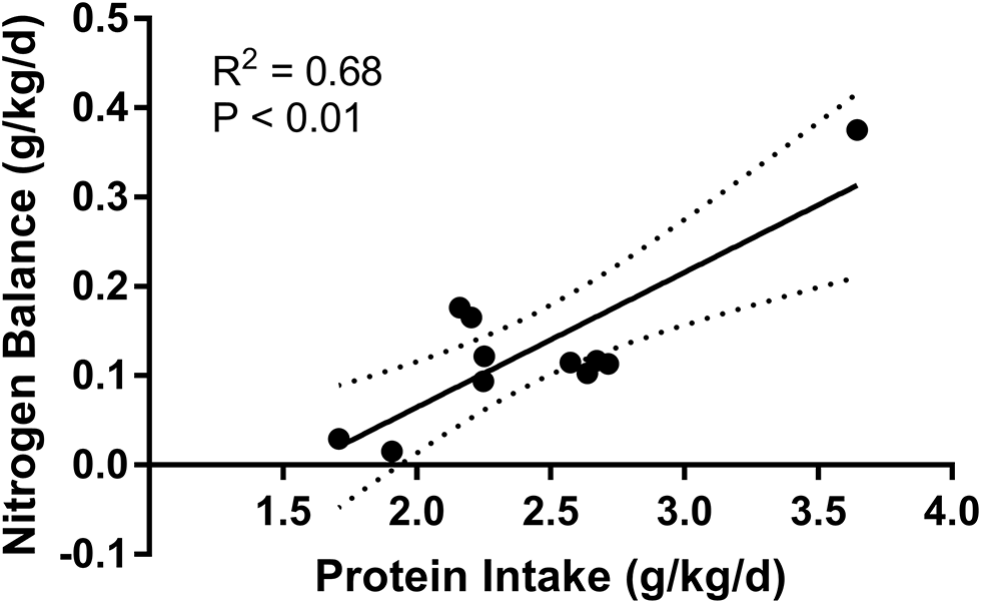
Relationship between nitrogen balance and protein intake. Each dot represents individuals. The solid line indicates the linear regression line of best fit, and the dashed lines represent the 95% confidence interval (CI). A significant positive correlation was observed (*R*^2^ =0.68, *p* < 0.01, *n* = 11).

The relationship between protein intake and NBAL is shown in Figure 1. A linear relationship was observed between the protein intake and NBAL (*R*^2^ = 0.68, *p* < 0.01). The estimated average protein intake for achieving zero NBAL was calculated to be 1.57 g/kg/day, and the population-safe protein intake was estimated to be 1.93 g/kg/day.

### Dietary Intake

**Figure 2:**
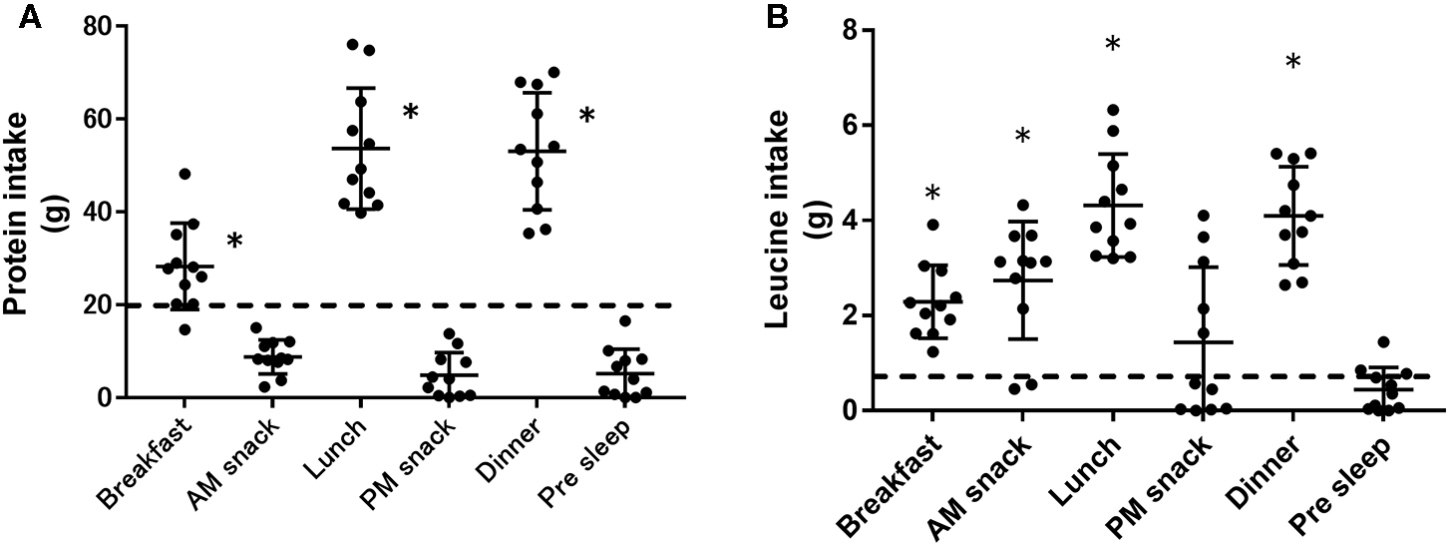
Distribution of the dietary protein and leucine consumed throughout day 2. The amount of protein or leucine consumed in each meal or snack is shown in **(A)** or **(B)**, respectively. The dashed line represents the recommended protein (20 g) **(A)** and leucine (0.7 g) **(B)** intake for each eating occasion. Data are shown as mean ± standard deviation (*n* = 11). Each dot represents individuals. *significant difference compared to the lower end of the recommended intake (20 g for protein and 0.7 g for leucine intake), respectively (15, 16).

Data on the dietary intake from days 1, 2, and 4 are shown in Table 1. The distribution and the composition of the dietary amino acids consumed throughout day 2 is shown in Figure 2. Protein intake (i.e., total amino acids intake) at breakfast, lunch, and dinner was higher than the lower end of the recommended intake of 20 g. Leucine intake in the three main meals was higher than the lower end of the recommended intake of 0.7 g. The leucine intake in the morning snack was higher than the lower end of the recommended intake of 0.7 g.

**Table 1:** Average daily energy intake and macronutrient breakdown.

	**Day 1**	**Day 2**	**Day 4**
**Energy**			
kcal/day	3394 ± 584	3455 ± 678	3250 ± 560
**Protein**			
g/day	163 ± 31	160 ± 28	156 ± 34
g/kg/day	2.5 ± 0.5	2.4 ± 0.5	2.4 ± 0.6
Energy %	19 ± 2	19 ± 2	19 ± 2
**Fat**			
g/day	112 ± 31	93 ± 25	91 ± 20
g/kg/day	1.7 ± 0.4	1.4 ± 0.4	1.4 ± 0.3
Energy %	30 ± 5	24 ± 5	25 ± 3
**Carbohydrate**			
g/day	431 ± 69	494 ± 105	451 ± 74
g/kg/day	6.5 ± 1.1	7.5 ± 2	6.9 ± 1.6
Energy %	51 ± 6	57 ± 5	56 ± 4

*Data are shown as mean ± standard deviation (n = 11)*.

### Energy Expenditure

The duration of handball practice sessions was 118.7 ± 0.0 min (on day 1), 119.6 ± 0.0 min, 102.0 ± 22.1 min (morning and afternoon on day 2), 121.4 ± 0.0 min (on day 4). HR and %HRmax during handball practice sessions was 133.6 ± 9.9 bpm and 69.3 ± 4.2 %HRmax (on day 1), 129.1 ± 8.4 bpm and 66.9 ± 3.7 %HRmax, 114.6 ± 9.1 bpm and 59.4 ± 4.8 %HRmax (morning and afternoon on day 2), 128.3 ± 11.4 bpm and 66.4 ± 5.0 %HRmax (on day 4). The total daily energy expenditure during the training camp was estimated to summarize the REE, DIT, and energy expenditure during habitual physical activity and during training sessions, as shown in Table 2.

**Table 2:** Energy expenditure during the training camp.

**Kcal**	**Day 1**	**Day 2**	**Day 4**
Resting energy expenditure	1,331 ± 112		
Diet-induced thermogenesis	241 ± 41	245 ± 48	231 ± 40
Energy expenditure during habitual physical activities	302 ± 87	268 ± 80	268 ± 64
Energy expenditure during training sessions	1,126 ± 116	1,478 ± 209	1,103 ± 143
Total energy expenditure	3,000 ± 228	3,322 ± 253	2,933 ± 208

*Data are shown as mean ± standard deviation (n = 11). Diet-induced thermogenesis was calculated as the total energy intake × 7.1% (18)*.

### Energy Balance

Positive energy balance (394±508 kcal) was observed on day 1, but there are no differences compared with zero on day 2 and 4 (133 ± 536 kcal, 318 ± 544 kcal, respectively).

## Discussion

The results of the current investigation of NBAL in elite female handball athletes during a national training camp suggested the estimated average and population-safe protein intake for maintaining zero NBAL to be 1.57 and 1.93 g/kg/day, respectively. A recent study demonstrated that the population-safe protein intake in team sports was 1.7 g/kg/day for female athletes (13), which is slightly lower than that of the current study. Possible reasons for this slight discrepancy include first, the current participants engaged in a larger training volume (i.e., 220 min of handball practice) compared to that in previous studies (100 min of simulated soccer match) (13, 14). On the other hands, although the intensity of exercise was not reported in the former study, the mean heart rate during the Loughborough Intermittent Shuttle Test, could be estimated 70% of maximum VO_2_ (23). Thus, the intensity of the exercise was similar with previous study. To sum up, due to longer exercise duration with similar intensity, the exercise volume of the current participants was higher compared to that in the previous study. Assuming that amino acid oxidation contributes ~5% of total energy expenditure during exercise (8), the exercise-induced oxidative loss of amino acid could lead to ~19 g of total protein or the equivalent of ~0.3 g/kg/day in the current study. On the other hand, in the previous study, the approximate energy expenditure during exercise was 735 kcal, which translates into ~9 g of total protein or the equivalent of ~0.13 g/kg/day (13). Thus, the large volume of exercise in the current study might lead to increased protein needs. Second, carbohydrate intake in the current participants might increase the protein requirements. While the current guideline recommends a carbohydrate intake of 8–12 g/kg/day for athletes engaging in 4–5 h/day of moderate-high intensity exercise (2), our participants consumed 7.3 g of carbohydrate/kg/day on day 2 when they performed for a total of 220 min. Low carbohydrate availability could increase the protein requirements (24), as low carbohydrate availability potentially increases the contribution of endogenous protein to energy production (8) and suppresses MPS (25). Thus, the low carbohydrate intake in the current study might lead to an increase in protein need. Finally, a different method was utilized to determine the protein requirements in the current study. While the NBAL technique was utilized in the current study because the can be used in a free-living condition, the indicator amino acid oxidation (IAAO) method was utilized in the previous study (13). While NBAL determines the protein intake achieving zero NBAL, the IAAO method reveals the protein intake for maximizing whole-body protein synthesis. The difference leads to values which are 30–50% greater than those based on NBAL data (9, 26). Assuming the protein requirement determined by NBAL technique underestimates 30–50% (9, 26), the protein requirement in the elite female handball athletes in this study might translate into 2.5–2.9 g/kg/day (1.9 g/kg/day, multiplied by 130–150%). On the other hand, it remains obscure whether the determined protein intake by NBAL technique can optimize athletic performance or body composition. Thus, further study is needed to validate whether the protein requirements in the current study can optimize exercise performance during training camp in elite female team sport athletes.

Recent reports indicated that the dose and timing of protein intake during the day could affect protein requirements. In the present study, protein intake in all three main meals (i.e., breakfast, lunch, and dinner) was higher than the 20 g recommended for maximizing MPS (15, 16). In addition, while an intake of 20 g of egg or whey protein maximizes the MPS after lower-limb resistance exercise (27, 28), 40 g of whey protein increased the MPS more than did 20 g of whey after whole-body resistance exercise (29). In the current study, the participants consumed more than 40 g of protein at lunch and dinner, which maximizes the MPS after whole-body resistance exercise. In contrast, protein ingestion immediately prior to sleep increases muscle protein synthesis during overnight sleep (30). Thus, adding pre-sleep protein ingestion to the dietary pattern may optimize the MPS throughout the day. In addition, in the present study, leucine intake was higher than the recommended amount in the morning snack as well as at the three main meals, but not in the afternoon and pre-sleep snacks, because some participants consumed a supplement containing essential amino acids with a high amount of leucine immediately after training sessions. Enriching the branched-chain amino acid intake in egg protein decreases the amino acid needs of endurance athletes (31). For these reasons, the consumption of amino acid supplements may lead to lower protein needs while optimizing MPS after exercise. Future studies focusing on the effect of the distribution and quality (i.e., amino acid composition) of the protein intake throughout the day on the protein requirements are warranted to determine the optimal pattern of protein and amino acid intake.

Our finding suggests that 1.9 g protein/kg/day is required to ensure a zero NBAL in 97.5% of the current participants. Previous studies investigating the habitual nutritional intake of female professional or elite athletes engaging in other team sports report a protein intake of 1.0–1.2 g/kg/day (4, 6, 32, 33). The discrepancy between the values and the current results indicates that some athletes consume less than the recommended protein intake (1.9 g/kg/day) evaluated in the present study. Since dietary surveys may underestimate nutritional intake (34, 35), further study assessing the nutritional status and physiological outcomes is needed to determine if these athletes consume sufficient amounts of protein.

Strength of the current study is to obtain unique findings regarding protein requirements of elite athletes who performed usual training. Since the information in elite athlete is limited owing to difficulties in conducting experiments with elite athletes, the results of the present study could be beneficial for determining the recommended protein intake in elite athletes. On the other hand, there are some limitations in the current study. First, the period of investigation was only a day. The current study utilized the NBAL technique to determine protein requirements in elite handball athletes in a free-living condition. However, protein requirements are generally estimated based on NBAL studies with at least two levels of protein intake for several days and up to 2 weeks (1, 11, 12). Thus, future studies with complete provision of all the foods for a longer period (i.e., several days up to 2 weeks) are needed to investigate whether the protein intake determined in the current study could achieve zero or positive nitrogen balance. Second, we could not recruit the targeted number (i.e. 20) of the participants due to lack of the overall participants in the training camps. However, we decided to stop recruitment because the participants who participated in the other training camp, are in different situation compared with the current participants. Although we found a significant relationship between protein intake and nitrogen balance, the current number of the participants might be underpowered, compared with the targeted number. Third, although the protein requirements were determined using the NBAL technique to identify the protein intake required for achieving zero NBAL, it remains obscure unclear whether the determined protein intake can optimize athletic performance or body composition. Thus, further study is needed to validate whether the protein requirements in the current study can optimize exercise performance during training camp in female elite team sport athletes.

In conclusion, the protein requirement for elite female handball athletes during training camp to achieve zero NBAL was 1.57 g/kg/day, and the population-safe protein intake was 1.93 g/kg/day. Since the dose and timing of protein and leucine intake were suboptimal throughout the day, the dietary pattern is possibly improved by the ingestion of protein or amino acids after exercise and before sleep.

## Data Availability Statement

The datasets generated for this study are available on request to the corresponding author.

## Ethics Statement

The studies involving human participants were reviewed and approved by the institutional review board of Ajinomoto Co., Inc. The participants provided their written informed consent to participate in this study.

## Author Contributions

This study was designed by HS, YU, TT, and HK. Data was collected by HS, YU, TT, and HK. Data analysis was performed by HS and HK. Data interpretation was performed by HS, YU, TT, and HK. The manuscript was drafted by HS, supervised by HK. All authors approved the final version of the manuscript.

## Conflict of Interest

The Japanese Handball Association received financial support from Ajinomoto Co., Inc. HS, YU, and HK are employed by Ajinomoto Co., Inc. The remaining authors declare that the research was conducted in the absence of any commercial or financial relationship that could be construed as a potential conflict of interest.

## Supplementary Material

Supplementary Figure 1 Schematics of the experimental protocol.
