# Supplementary material for: Nitrogen Balance in Female Japanese National Handball Players During Training Camp
Source: Front Nutr. 2020 May 12;7:59. doi: 10.3389/fnut.2020.00059 (PMC7236612; doi:10.3389/fnut.2020.00059)
Supplement: Supplementary Figure 1 — Schematics of the experimental protocol. [file Data_Sheet_1.PDF]

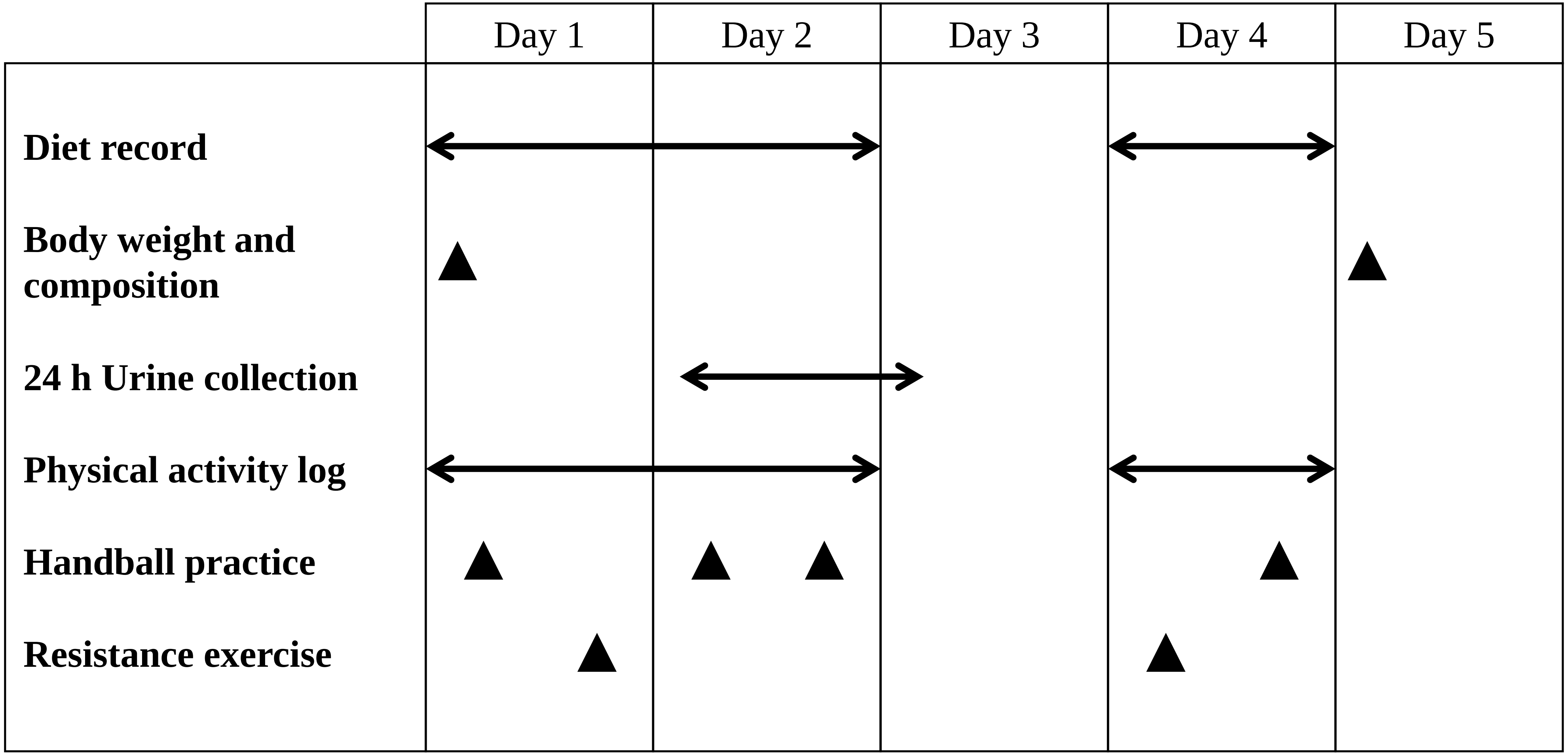
Day 1
Day 2
Day 3
Day 4
Day 5
Diet record
Body weight and composition
24 h Urine collection
Physical activity log
Handball practice
Resistance exercise
